# Alterations in arterial function after high-voltage electrical injury

**DOI:** 10.1186/cc11190

**Published:** 2012-02-12

**Authors:** Kyoung-Ha Park, Woo Jung Park, Min-Kyu Kim, Hyun-Sook Kim, Seong Hwan Kim, Goo-Yeong Cho, Young-Jin Choi

**Affiliations:** 1 Department of Cardiology, Hallym University Hangang Sacred Heart Hospital, 94-200, Yeongdeungpo-dong, 150-030 Seoul, Korea; 2 Department of Cardiology, Hallym University Sacred Heart Hospital, 896, Yeongdeungpo-dong, Pyeongchon-dong, Dongan-gu Anyang, 431-070 Gyeonggi-do, Korea; 3 Department of Cardiology, Korea University Ansan Hospital, Cojan 1-Dong, Danwon-Gu, Ansan-Si, 152-703 Gyeonggi-do, Korea; 4 Department of Cardiology, Seoul National University Hospital, Gumi-Ro 173, Bundang-Gu, Seongnam-Si, 463-707 Gyeonggi-do, Korea

**Keywords:** high-voltage electrical injury, endothelium, smooth muscle, arterial function, flow-mediated dilation, nitrate-mediated dilation

## Abstract

**Introduction:**

The aim of this study was to evaluate the functional changes of the arterial endothelium and smooth muscle after a high-voltage electrical injury (HVEI), using flow-mediated dilation (FMD) and nitrate-mediated dilation (NMD).

**Methods:**

Twenty-five male patients injured in the upper extremities by current due to contact with more than 20,000 volts were enrolled in the study. FMD and NMD were measured on the brachial artery within 48 hours after HVEI, and follow-up FMD and NMD were evaluated six weeks later. In addition, we enrolled an age, sex and body mass index matched healthy control group consisting of 25 individuals. Including FMD and NMD, all the variables of the control group were investigated one time and compared with the initial and six week follow-up data of the HVEI group.

**Results:**

A significantly lower initial FMD was seen in the HVEI group compared with the control group (2.1 ± 1.2% versus 13.6 ± 3.4%, *P *< 0.01). At the six week follow-up, the FMD of the HVEI group had significantly improved compared to the initial FMD (2.1 ± 1.2% versus 5.1 ± 2.1%, *P *< 0.01), but it was still lower than the FMD of the control group (5.1 ± 2.1% versus 13.6 ± 3.4%, *P *< 0.01). A significantly lower NMD was seen both initially and at the six week follow-up compared with the NMD of the control group (7.3 ± 4.7% versus 20.4 ± 4.1%, *P *< 0.01 and 11.4 ± 6.7% versus 20.4 ± 4.1%, *P *< 0.01, respectively). The FMD study of the contralateral arm which was uninjured by HVEI was available in six patients. In those patients, the six week follow-up FMD was significantly improved in the HVEI arm compared with the initial FMD (1.8 ± 0.6% versus 4.4 ± 1.6%, *P *< 0.01). However, in the contralateral uninjured arm, there was no difference between the initial and the six week follow-up FMDs (5.5 ± 1.4% versus 6.9 ± 2.2%, *P *= 0.26).

**Conclusions:**

After HVEI, the endothelial and smooth muscle functions of the brachial artery were significantly decreased for at least six weeks. Long term cautious care might be needed for all victims of HVEI, because there is a chance of increased risk of thrombosis or stenosis in the injured arm.

## Introduction

High-voltage electrical injury (HVEI) is defined as exposure to more than 1,000 volts and it causes devastating local and systemic damage [1]. Usually, HVEI is associated with entry and exit wounds, and assessment of the pathway allows prediction of the organs at most risk of damage [2]. In HVEI, nearly 90% of victims have injuries in their upper extremities [3,4], with amputation required in 24% to 49% of cases [5,6]. The extent of the injury depends on the electrical voltage, the local tissue resistance, the pathway of the current flow, and the duration of the contact [7]. The severity of the damage to the arteries is also a critical factor [8]. In a mouse femoral artery model of extensive electrical injury, Carmeliet *et al. *showed that the recovery process of the endothelium and smooth muscle was quickly initiated and reached a peak two weeks after injury [9]. However, the functional changes that occur in human arteries after HVEI have not yet been studied. Therefore, the purpose of the present study was to evaluate the functional changes of the arterial endothelium and smooth muscle after HVEI using flow-mediated dilation (FMD) and nitrate-mediated dilation (NMD).

## Materials and methods

### Study population

Patients were eligible for enrollment in this study if they were over 18 and under 65 years of age, injured in the upper extremities, and had undergone HVEI of more than 20,000 volts in an upper extremity. Patients were excluded if they were injured more than 48 hours before the study; had any cardiovascular disease, diabetes, renal insufficiency, or left ventricular dysfunction (LVEF < 55%); had no HVEI in an upper extremity; had serious external wounds or ischemic changes in the injured upper extremity; had sepsis and systemic shock; or if they were unable to follow the protocol. In addition, we enrolled an age, sex, and body mass index matched healthy control group to compare the data of the patients with HVEI.

### Measurement of the FMD and NMD

The patients in this study, whose arms had been injured by high-voltage electrical current, were evaluated using FMD and NMD. An experienced vascular sonographer performed an ultrasound examination using a Vivid 7 ultrasound system (GE Vingmed Ultrasound, Horten, Norway) with a 12 MHz linear array transducer. The baseline study was conducted within 48 hours after HVEI and the follow-up study was conducted six weeks after the initial study. FMD and NMD were measured according to the recommendations of Coretti and colleagues [10]. In brief, patients were told not to exercise, not to ingest substances that might affect FMD and NMD, such as caffeine, foods, or vasoactive medication, and not to use tobacco for at least 12 hours before the study. A landmark was chosen 5 cm proximal to the antecubital crease and the brachial artery (BA) was imaged. The baseline diameter of the BA was measured from 2-dimensional gray scale longitudinal images. Subsequently, a blood pressure cuff was inflated at the distal forearm up to 220 mmHg for five minutes. After cuff release, the BA diameter was measured again at 40, 60, 80, and 90 seconds. Ten minutes after taking the measurements, an exogenous nitric oxide donor, sublingual nitroglycerin (0.6 mg), was administered. Three minutes after that, images were recorded for NMD measurements. All images were recorded digitally by capturing the BA in the longitudinal plane with an electrocardiogram. The BA diameter image for analysis was chosen at the onset of the R-wave on the electrocardiogram. Measurements were performed at seven points, and the highest and lowest values were discarded. The mean value from the remaining five measurements was used for further analysis. The follow-up FMD and NMD were measured six weeks later. Including the FMD and NMD, all of the control group variables were investigated only one time. One cardiologist (WJP), who was blinded to the participants' clinical data, interpreted the ultrasound results using an off-line method.

### Echocardiographic assessment

One experienced sonographer, who was blinded to the patients' information, performed the echocardiography. The two-dimensional (2D) M-mode image was recorded using an echocardiography machine (Vivid 7; GE Medical Systems, Milwaukee, USA) according to the guidelines of the American Society of Echocardiology [11]. The left ventricular ejection fraction was quantified by Simpson's rule using the 2D echocardiography images from the apical four-chamber view [12]. Measurements were made on-line and recorded digitally with participants' initials and study number as their only forms of identification. One cardiologist (MKK), blinded to the participant's clinical data, interpreted the echocardiogram using an off-line method.

### Statistical analysis

Data are presented as mean ± standard deviation (SD). Comparisons of data across the two time points (within 48 hours after injury and six weeks after injury) were performed using Student's 2-tailed, paired *t *test. Comparisons were made between the HVEI group and the control group using either an independent samples *t *test or a Mann Whitney U test. Differences in categorical variables between the two groups were analyzed with either the Chi-square test or Fisher's exact test. Statistical analyses were performed using SPSS version 17.0 (SPSS Inc., Chicago, IL, USA). All probability values were two-sided. A value of *P *< 0.05 indicated statistical significance. This study was approved by the Institutional Review Board of the Hallym University Medical Center (IRB No. 2009-062) and all patients gave their written informed consent.

## Results

Between February 2010 and April 2011, 94 victims of HVEI were assessed. Most of them were electrical engineers who were injured while repairing an industrial electrical transformer (*n *= 56) or power pole (*n *= 21). The rest were injured while working at a drainage pump station (*n *= 10), doing sign work (*n *= 3), or landscaping work (*n *= 2), and so on. Among the screened victims, 69 patients were excluded for the following reasons: serious injury to both arms (*n *= 36), admitted more than 48 hours after the event (*n *= 12), were without electrical injury in an upper extremity (*n *= 7), multi-organ failure or sepsis (*n *= 6), combined serious flame burns on both arms (*n *= 4), diabetes (*n *= 2), or refused to participate in the study (*n *= 2). Among the 25 enrolled HVEI patients, nine had unilateral upper extremity HVEI. The mean value of the serum creatinine kinase was 5,133 ± 7,716 IU/L and myoglobinuria was detected in 84% (21 out of 25) of the studied patients. During the study, two patients did not undergo the six week follow-up FMD and NMD because arterial thrombosis and obstruction led to musculocutaneous flap failure and amputation of the distal injured arm in the second and third weeks after HVEI.

### Baseline patient characteristics

No significant differences were noted between the HVEI group and the control group in terms of baseline clinical characteristics including age, body mass index, cardiovascular risk factors, and medications used, except for elevated fasting glucose and high-sensitive C-reactive protein in the HVEI group (Table 1).

**Table 1:** Baseline Clinical Characteristics and Medications

	HVEI group (*n *= 25)	Control group (*n *= 25)	*P*-Value
Age (years)	48 ± 11	48 ± 11	0.94
Hypertension	6 (24%)	7 (28%)	0.75
Current smoking	14 (56%)	9 (36%)	0.26
Body mass index (Kg/m^2^)	23.6 ± 2.6	23.6 ± 2.0	0.99
Hemoglobin (gm/dL)	14.7 ± 1.1	14.9 ± 1.1	0.62
Glucose (mg/dL)	110 ± 18	90 ± 11	< 0.01
Total cholesterol (mg/dL)	175 ± 27	185 ± 26	0.18
Creatinine (mg/dL)	0.8 ± 0.2	0.8 ± 0.1	0.65
High-sensitive C-reactive protein (mg/dL)	51.2 ± 43.2	0.9 ± 0.6	< 0.01
Aspirin	2 (8%)	5 (20%)	0.42
Beta-blocker	1 (4%)	2 (8%)	1.00
ACEI/ARB	2 (8%)	5 (20%)	0.42
Calcium channel blocker	6 (24%)	3 (12%)	0.46
Statin	1 (4%)	4 (16%)	0.35

ACEI/ARB, angiotensin converting enzyme inhibitor/angiotensin receptor blocker; HVEI, high-voltage electrical injury.

### Changes in clinical parameters and arterial function

The changes in the clinical parameters and arterial functions are shown in Table 2. The initial systolic and diastolic blood pressure and heart rate were higher in the HVEI group than in the control group, but no difference was observed at the six week follow-up. The BA was significantly enlarged during the initial study in the HVEI group compared with the control group (4.4 ± 0.6 mm versus 3.9 ± 0.4 mm, *P *< 0.01). However, no difference was seen in the size of the BA between the HVEI group and the control group at the six week follow-up (4.1 ± 0.5 mm versus 3.9 ± 0.4 mm, *P *= 0.07). During the study period, no difference was observed in left ventricular function between the HVEI group and the control group.

**Table 2:** Influence of high voltage electrical injury on clinical parameters and arterial functions

	Control group (*n *= 25)	HVEI group	
		
		< 48 hrs (*n *= 25)	6 Weeks (*n *= 23)
Systolic blood pressure (mmHg)	120 ± 14	146 ± 14^a^	120 ± 8
Diastolic blood pressure (mmHg)	71 ± 13	84 ± 13^a^	76 ± 7
Heart rate (beats/min)	72 ± 8	83 ± 10^a^	74 ± 4
LVEF (%)	64 ± 4	66 ± 3	66 ± 5
Size of brachial artery (mm)	3.9 ± 0.4	4.4 ± 0.6^a^	4.2 ± 0.5

^a^*P *value < 0.01 versus control. HVEI, high-voltage electrical injury; LVEF, left ventricular ejection fraction.

### Changes in FMD and NMD

**Figure 1:**
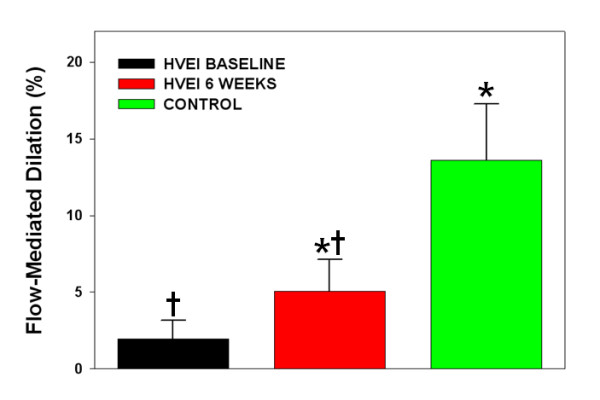
**Changes in FMD across the study compared with the control group**. The initial FMD was significantly lower in the HVEI group (*n *= 25) than in the control group (*n *= 25; 2.1% ± 1.2% versus 13.6% ± 3.4%, *P *< 0.01). At the six week follow-up, the FMD was improved in the HVEI group compared with the initial FMD (2.1% ± 1.2% versus 5.1% ± 2.1%, *P *< 0.01) but it was still lower than the FMD of the control group (5.1% ± 2.1% versus 13.6% ± 3.4%, *P *< 0.01). *P < 0.01 versus baseline and †*P *< 0.01 versus control group. FMD, flow-mediated dilation; HVEI, high-voltage electrical injury.

**Figure 2:**
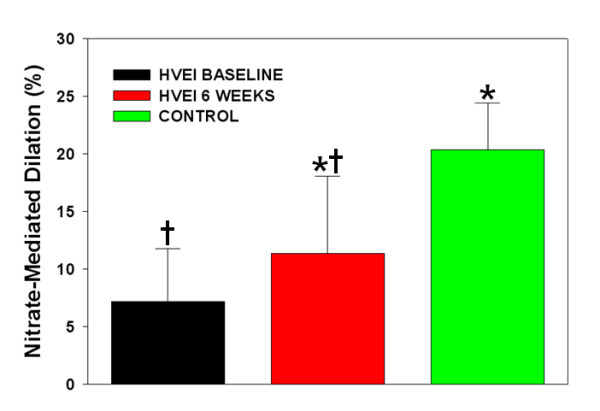
**Changes in NMD across the study compared with the control group**. The initial NMD was significantly lower in the HVEI group (*n *= 25) than in the control group (*n *= 25; 7.3% ± 4.7% versus 20.4% ± 4.1%, *P *< 0.01). At the six week follow-up, the NMD was improved in the HVEI group compared with the initial NMD (7.3% ± 4.7% versus 11.4% ± 6.7%, *P *< 0.01) but it was still lower than the NMD of the control group (11.4% ± 6.7% versus 20.4% ± 4.1%, *P *< 0.01).*P < 0.01 versus baseline and †*P *< 0.01 versus control group. HVEI, high-voltage electrical injury; NMD, nitrate-mediated dilation.

**Figure 3:**
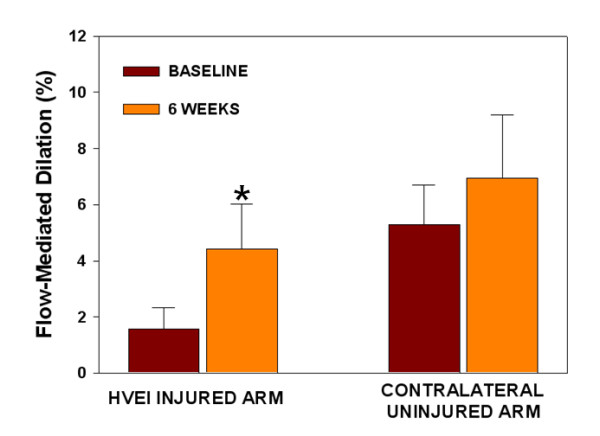
**Changes in FMD of the unilateral upper extremity HVEI compared with the contralateral uninjured arm in six patients**. The six week follow-up FMD was significantly improved in the HVEI arm compared with the initial FMD (1.8% ± 0.6% versus 4.4% ± 1.6%, *P *< 0.01). However, in the contralateral uninjured arm, there was no difference between FMDs in the initial study and at the six week follow-up (5.5% ± 1.4% versus 6.9% ± 2.2%, *P *= 0.26). In addition, the six week follow-up FMD of the contralateral injured arm was significantly lower than the matched control (6.9% ± 2.2% versus 13.6% ± 4.4%, *P *< 0.01).*P < 0.01 versus baseline; FMD, flow-mediated dilation; HVEI, high-voltage electrical injury.

The initial FMD was significantly lower in the HVEI group than in the control group (2.1 ± 1.2% versus 13.6 ± 3.4%, *P *< 0.01, Figure 1). At the six week follow-up, the FMD was significantly improved in the HVEI group compared with the initial FMD (2.1 ± 1.2% versus 5.1 ± 2.1%, *P *< 0.01), but it was still lower than the FMD of the control group (5.1 ± 2.1% versus 13.6 ± 3.4%, *P *< 0.01). The six week follow-up NMD improved in the HVEI group compared with the initial NMD (7.3 ± 4.7% versus 11.4 ± 6.7%, *P *< 0.01, Figure 2). However, both the initial and the six week follow-up NMDs were significantly lower in the HVEI group than in the control group (7.3 ± 4.7% versus 20.4 ± 4.1%, *P *< 0.01 and 11.4 ± 6.7% versus 20.4 ± 4.1%, *P *< 0.01, respectively). Among the 25 enrolled patients, nine had unilateral upper extremity HVEI. The contralateral arm was evaluated for the study in six out of those nine patients, because three patients suffered from severe contralateral axillary wounds as exit-point injuries, which may affect the flow of the brachial artery and influence the FMD results of the contralateral uninjured arm. The FMD changes in the six patients are presented in Figure 3. At the six week follow-up, the FMD was significantly improved in the HVEI arm compared with the initial FMD (1.8 ± 0.6% versus 4.4 ± 1.6%, *P *< 0.01). However, in the contralateral uninjured arm, there was no difference between FMDs in the initial study and at the six week follow-up (5.5 ± 1.4% versus 6.9 ± 2.2%, *P *= 0.26). In addition, the six week follow-up FMD of the contralateral injured arm was significantly lower than the matched control (6.9 ± 2.2% versus 13.6 ± 4.4%, *P *< 0.01).

## Discussion

The principal finding of this study was that, following HVEI, the endothelial and smooth muscle functions of the injured artery were significantly decreased even after six weeks, compared to the arterial functions of the healthy controls.

The human body is a good electrical conductor and the systemic effects and tissue damage are directly proportional to the magnitude of the current drawn by the victim [1]. These injuries can result in life-threatening complications, such as respiratory arrest, ventricular fibrillation and acute renal failure [13]. Many cases of HVEI to the upper arms inevitably lead to amputation because the massively destroyed vessel walls easily undergo coagulation necrosis, leading to thrombosis and local obstruction of blood flow. The distal ends of the injured limbs may then become gangrenous [5,14].

In the animal model, a more severe injury was induced in the vessel wall by an electric current than would occur from a mechanical injury [9,15]. With the mouse femoral artery electrical injury model, the endothelial cells started to proliferate immediately after injury and proliferated very actively during the first two weeks. In the first week after injury, the media and neointima both contained a heterogeneous mix of smooth muscle cells and leukocytes, which proliferated actively. The majority of the cells in the media and neointima had proliferated maximally by two weeks after injury and had become more quiescent by four weeks [9,16,17]. In the present study, the FMD and NMD values at six weeks were still lower than the control group values. This means that the cellular process is essentially complete in four weeks [9] and yet functional abnormality persists for at least six weeks (maybe longer). Therefore, there seems little prospect of improvement in histological tissue repair and this may be relevant to the longevity of the disabling symptoms seen. This result indicates that the approaches to lessen the damage and support the recovery of the arterial endothelium and smooth muscle should be initiated as rapidly as possible after an electrical injury. In our opinion, anti-oxidants or nitric oxide favoring agents, such as vitamin E, L-arginine, or trimetazidine could be considered [18,19]. If the victim has hypertension, an angiotensin converting enzyme inhibitor, angiotensin receptor antagonist, or nebivolol might be a beneficial antihypertensive agent to the endothelium [20].

HVEI to the upper extremities may result in amputation or graft surgery, although some patients are able to use their arms or legs without major disability if they do not have severe vascular damage or major tissue loss. Unfortunately, however, little information exists regarding the process and prognosis involved in the vascular damage caused by HVEI in humans. The present study showed that continuous impairment of the brachial arterial function occurred that involved the endothelium and smooth muscle, even if the victim did not have serious tissue damage or upper extremity loss. This finding means that long-term cautious care is needed for all victims of HVEI, as they have an increased risk of thrombosis or stenosis of the artery in the injured arm. In this study, two patients were not examined by FMD and NMD at the six week follow-up because of musculocutaneous flap failure and amputation of their distal injured arms, which might have been caused by insufficient arterial supply due to thrombosis and obstruction of the injured artery. In patients with severe high-voltage injury, Urich *et al. *showed that clotting activation and hypo-fibrinolysis led to progressive tissue necrosis and delayed arterial thrombosis [21]. In addition, a recent study showed that the reconstruction of tissue defects after an electrical trauma is associated with higher complication rates because of thrombus formation in injured arteries [22].

In this study, nine patients had unilateral upper extremity HVEI and three of the nine patients suffered from severe contralateral axillar area wounds as exit-point injuries, which may involve the axillary artery. The injured axillary artery may affect the distal blood flow of the distal brachial artery and influence the results of the FMD and NMD of the contralateral uninjured arm. Therefore, the FMD of the contralateral arm was evaluated in six patients. In these patients, the six week follow-up FMD was significantly improved in the HVEI arm compared with the baseline FMD. In the contralateral uninjured arm, there was no significant improvement in the six week FMD compared with the baseline FMD and the six week FMD was significantly lower than the normal control. This means that although the contralateral arm was not directly injured by the high voltage current, there might be systemic effects affecting areas that are distant from the direct current passage and the impairment of endothelial function may continue.

This study has several potential limitations. First, HVEI usually causes extensive arterial injuries associated with occlusive thrombosis, aneurysm formation and vessel wall rupture. In this study, 45% of the HVEI patients were not included due to the seriousness of their arm injuries or systemic damage (*n *= 42) observed during the screening. Therefore, there was a possibility that only patients with less severe tissue damage and maintained arterial flow would be enrolled in the study. Second, FMD may be influenced, to varying degrees, by many factors such as sympathetic tone, medications, and caffeine use. In addition, during the initial FMD study after HVEI, the patients might have been in an especially stressed condition, with increased sympathetic tone, than at the six week follow-up FMD, which might have decreased the initial FMD value [23]. Third, after HVEI, the integrity of the endothelium is crucial for physiologic vascular function. With increasing endothelial dysfunction, uncontrolled clotting activation and ischemia are initiated. This, in turn, enhances a vicious cycle, leading to multiple organ failure and death. Therefore, biomarkers reflecting this special compartment, such as the von Willebrand factor, asymmetric dimethyl arginine, angiopoietin 1 and 2, and the vascular endothelial growth factor may help in the early detection of local and systemic endothelial dysfunction and its complications after HVEI, but we did not evaluate this issue [24]. Fourth, the last follow-up FMD and NMD were measured six weeks after HVEI and it remains unanswered whether different results would have been obtained with an examination at a later follow-up date.

## Conclusions

In conclusion, after HVEI, the endothelial and smooth muscle functions of the brachial artery were significantly decreased for at least six weeks compared to the arterial functions of control patients. Long-term cautious care is needed when treating all victims of HVEI, as they might be at increased risk for thrombosis and stenosis of the arteries of the injured arm.

## Key messages

● The endothelial and smooth muscle functions of the brachial artery were significantly decreased for at least six weeks after a high-voltage electrical injury.

● Although the contralateral arm was not directly injured by the high voltage current, there might be effects on endothelial dysfunction caused by the high-voltage electrical injury and the impairment of the endothelial function may continue.

● Long-term cautious care is needed when treating all victims of high-voltage electrical injury, as they might be at increased risk of thrombosis and stenosis of the arteries of the injured arm.

## Abbreviations

BA: brachial artery; FMD: flow-mediated dilation; HVEI: high-voltage electrical injury; NMD: nitrate-mediated dilation.

## Competing interests

The authors declare that they have no competing interests.

## Authors' contributions

KHP and WJP conceived and designed the study; KHP, WJP and SHK analyzed and interpreted the data; KHP, SHK, GYC and HSK drafted the manuscript; KHP, GYC and YJC critically revised the manuscript for important intellectual content; MKK and WJP acquired the data. All authors read and approved the final manuscript for publication.
